# SARS-CoV-2 Infected Cardiomyocytes Recruit Monocytes by Secreting CCL2

**DOI:** 10.21203/rs.3.rs-94634/v1

**Published:** 2020-11-17

**Authors:** Shuibing Chen, Liuliu Yang, Benjamin Nilsson-Payant, Yuling Han, Fabrice Jaffré, Jiajun Zhu, Pengfei Wang, Tuo Zhang, David Redmond, Sean Houghton, Rasmus Møller, Daisy Hoagland, Shu Horiuchi, Joshua Acklin, Jean Lim, Yaron Bram, Chanel Richardson, Vasuretha Chandar, Alain Borczuk, Yaoxing Huang, Jenny Xiang, David Ho, Robert Schwartz, Benjamin tenOever, Todd Evans

## Abstract

Heart injury has been reported in up to 20% of COVID-19 patients, yet the cause of myocardial histopathology remains unknown. In order to study the cause of myocardial pathology in COVID-19 patients, we used a hamster model to determine whether following infection SARS-CoV-2, the causative agent of COVID-19, can be detected in heart tissues. Here, we clearly demonstrate that viral RNA and nucleocapsid protein is present in cardiomyocytes in the hearts of infected hamsters. Interestingly, functional cardiomyocyte associated gene expression was decreased in infected hamster hearts, corresponding to an increase in reactive oxygen species (ROS). This data using an animal model was further validated using autopsy heart samples of COVID-19 patients. Moreover, we show that both human pluripotent stem cell-derived cardiomyocytes (hPSC-derived CMs) and adult cardiomyocytes (CMs) can be infected by SARS-CoV-2 and that CCL2 is secreted upon SARS-CoV-2 infection, leading to monocyte recruitment. Increased CCL2 expression and macrophage infiltration was also observed in the hearts of infected hamsters. Using single cell RNA-seq, we also show that macrophages are able to decrease SARS-CoV-2 infection of CMs. Overall, our study provides direct evidence that SARS-CoV-2 infects CMs in vivo and proposes a mechanism of immune-cell infiltration and pathology in heart tissue of COVID-19 patients.

### Introduction

Respiratory failure is the predominant outcome in the ongoing Coronavirus Disease 2019 (COVID-19) pandemic, yet cardiac involvement is a common feature in hospitalized COVID-19 patients and is associated with worse disease outcomes. In fact, reports have shown that the mortality risk associated with acute cardiac injury is more significant than other common risk factors such as age, chronic pulmonary disease or prior history of cardiovascular disease[1, 2]. For example, in a Wuhan cohort, 7% of total patients and 22% of critically ill patients suffered myocardial injury, evidenced by elevated cardiac biomarkers, such as high sensitivity Troponin I (hs-cTnI) or by electrocardiography (ECG) and echocardiogram abnormalities[3]. Hs-cTnI was reported to be above the 99th percentile upper reference in 46% of non-survivors as opposed to 1% of survivors[4]. In addition, increasing numbers of cases of COVID-19 related Kawasaki disease-like symptoms are reported in children[5]. There are also several case reports of myocarditis in COVID-19 patients[6-8]. It is still unknown how cardiac injury is caused in COVID-19, but potential mechanisms could involve increased cardiac stress due to respiratory failure and hypoxemia, direct myocardial infection by SARS-CoV-2, or indirect cardiotoxicity from a systemic inflammatory response.

We and other groups have reported SARS-CoV-2 infection *in vitro* in human pluripotent stem cell-derived cardiomyocytes (hPSC-CMs)[9-11]. Although several studies have detected viral RNA in heart tissues from autopsies of COVID-19 patients[12, 13], it remains controversial whether SARS-CoV-2 can be found in cardiomyocytes (CMs). However, viral particles have been identified in interstitial cells of the myocardium of COVID-19 patients[7,13]. Interestingly, SARS-CoV-2 virions were detected in cardiac tissues of an 11-year-old child with multisystem inflammatory syndrome in children, a serious condition associated with COVID-19, who developed cardiac failure and passed way one day after being hospitalized[14].

Another potential cause of cardiac pathogenesis could be immune cell mediated tissue damage. Despite the controversy around SARS-CoV-2 infection of CMs, several studies using COVID-19 post-mortem heart samples consistently identified abnormal inflammatory infiltrates composed of $CD11b^{+}$macrophages[12], $CD68^{+}$ macrophages[7, 13], and, to a lesser extent, T cells[15], supporting the idea that immune cells could be the cause of cardiac injuries seen in COVID-19 patients.

Considering that most autopsy samples were collected several weeks after acute SARS-CoV-2 infection and that the only autopsy samples where viral particles were detected in cardiac tissues were taken from a patient one day after hospitalization, we hypothesize that the timing of sample collection is critical to detect SARS-CoV-2 virions in cardiac tissues. Due to the challenges of collecting heart biopsies from COVID-19 patients after or during acute infection, we used hamsters to mimic COVID-19 in an animal model and to systematically examine the role of SARS-CoV-2 in the pathology of heart tissues. Here, we show evidence of SARS-CoV-2 infection of hearts of infected hamsters. Both viral RNA and viral nucleocapsid protein was detected in CMs of acutely infected hamsters. Furthermore, we demonstrate that infection corresponded to decreased expression of CM markers and an increase of reactive oxygen species (ROS), which we further validated in autopsy heart samples from COVID-19 patients. In addition, we set up an immuno-cardiac co-culture platform using hPSC-derived CMs and monocytes/macrophages and found that CMs recruit monocytes by secretion of CCL2. Interestingly, we also showed that macrophages are able to inhibit SARS-CoV-2 infection of CMs. Together, we provided robust evidence of SARS-

CoV-2 infection of CMs *in vivo* and created an hPSC-based platform to model immune cell infiltration in hearts of COVID-19 patients.

### RESULTS

#### SARS-CoV-2 is detected in the cardiomyocytes of SARS-CoV-2 infected hamsters.

As studying the role of SARS-CoV-2 in cardiac pathology in COVID-19 patients is difficult, we instead utilized Syrian Golden Hamsters (*Mesocricetus auratus*), which are naturally susceptible to SARS-CoV-2 infection and develop a respiratory disease akin to COVID-19 [16, 17]. In order to investigate whether hamsters show any cardiopathology and infection of cardiac tissues after exposure to SARS-CoV-2, we intranasally infected hamsters with SARS-CoV-2. Two days post-infection (dpi), hearts were collected and separated into left ventricle (LV), left atrial (LA), right atrial (RA) and right ventricle (RV) before further analysis. Interestingly, bulk RNA-seq analysis of the collected hearts showed that transcripts covering most of the viral genome were detected in the LA, LV, RA, but not RV of infected hamsters (**Fig. 1a**). Strengthening these observations, we were also able to detect viral nucleocapsid (N) protein staining in CMs. Principal component analysis (PCA) showed that LV tissues of mock and SARS-CoV-2 hamsters clustered separately (**Fig. 1c**). Ingenuity pathways analysis (IPA) of genes that are significantly changed in mock versus SARS-CoV-2 infected LV tissues highlighted Coronavirus Pathogenesis Pathway and Production of Nitric Oxide and Reactive Oxygen Species in Macrophages pathways (**Fig. 1d**). Heatmaps showed the downregulation of functional CM associated genes (**Fig. 1e**) and the upregulation of ROS related genes (**Fig. 1f**). We further analyzed the transcript profiles from heart autopsies from 5 healthy donors and 3 COVID-19 patients. Consistent with the data of SARS-CoV-2 infected hamster hearts, the cardiac tissues of COVID-19 patient hearts showed decreased expression of functional CM associated genes and increased expression of ROS associated genes (**Fig. 1g, 1h**). Together, these data provide the first evidence of SARS-CoV-2 infection of CMs in an *in vivo* animal model. In addition, we observed markers indicating cardiac injury both in hamsters and in clinical COVID-19 samples, suggesting that SARS-CoV-2 infection can cause CM damage.

**SARS-CoV-2 infected cardiomyocytes secrete CCL2.**

Our previous studies showed that hPSC-derived CMs are permissive to SARS-CoV-2 infection[10], and established a platform to model response of CMs to SARS-CoV-2 infection. CMs were derived from an MYH6:mCherry H9 hESC reporter line or a human iPSC line[18] (**Extended Data Fig. 1a**). Over 90% of the cells expressed mCherry and/or stained positive with antibodies recognizing sarcomeric α-actinin and cTNT (**Extended Data Fig. 1b**).

The H9 hESC-derived CMs were infected with SARS-CoV-2 (USA-WA1/2020) (MOI=0.1). qRT-PCR using primers targeting N subgenomic RNA transcripts detected replicating viral RNA at 24 hours post infection (hpi) (**Fig. 2a**). Plaque assays further validated the production of infectious virus in the supernatant at 24 hpi (**Fig. 2b**). Transcript profiling comparing mock and SARS-CoV-2 infected H9 hESC-derived CMs aligning transcripts with the viral genome confirmed robust viral replication in H9 hESC-derived CMs (**Fig. 2c**). Immunostaining assays further confirmed the infection of CMs by SARS-CoV-2 (**Fig. 2d**). PCA and clustering analysis showed that RNA profiles from mock and SARS-CoV-2 infected H9 hESC-derived CMs clustered separately (**Fig. 2e, 2f**). The volcano plot and heatmap revealed robust induction of chemokines in infected H9 hESC-derived CMs, including *CCL2* (**Fig. 2g, 2h**). KEGG pathway analysis of differentially expressed genes highlighted pathways involved in inflammatory and immune responses, including TNF signaling pathway, cytokine-cytokine receptor interaction, NF-kappa B signaling pathway, and IL-17 signaling pathway (**Fig. 2i**).

We further examined the response of adult human CMs to SARS-CoV-2 infection. Adult human CMs were infected with SARS-CoV-2 (USA-WA1/2020, MOI=0.1). Similar to hPSC-derived CMs, significant levels of viral subgenomic RNA (**Fig. 2j**) and robust read coverage across the viral genome (**Fig. 2k**) were detected in adult human CMs. PCA and clustering analysis showed that mock and SARS-CoV-2 infected adult human CM transcript profiles clustered separately (**Fig. 2l, 2m**). Furthermore, consistent with data from hPSC-derived CMs, analysis of the host transcriptional response revealed robust induction of chemokines, including *CCL2* (**Fig. 2n, 2o**). Consistent with hPSC-derived CMs (**Fig. 2i**), KEGG pathway analysis in adult human CMs highlighted pathways involved in inflammatory and immune responses, including IL-17 signaling pathway, TNF signaling pathway, cytokine-cytokine receptor interaction, and chemokine signaling pathway (**Fig. 2p**). Finally, ELISA assays confirmed significantly increased levels of CCL2 in the medium of H9 hESC-derived CMs after SARS-CoV-2 infection, compared to mock infected cells (**Fig. 2q**).

**CCL2 and macrophage infiltration are detected in hearts of SARS-CoV-2 infected hamsters.**

In order to test whether CCL2 secretion upon SARS-CoV-2 can also be detected *in vivo*, we further examined CCL2 expression levels in the hearts of SARS-CoV-2 infected hamsters. Consistent with SARS-CoV-2 our findings in infected hPSC-derived CMs, the LA, LV, and RA of SARS-CoV-2 infected hamsters showed increased levels of *CCL2* (**Fig. 3a**), which was further validated by immunostaining (**Fig. 3b, 3c**). Cell-mixture deconvolution using LM22 matrix[19] identified the enrichment of pro-inflammatory macrophages in the LA, LV and RA of SARS-CoV-2 infected hamsters (**Fig. 3d**), which is consistent with previous reports of abnormal macrophage infiltration in hearts of COVID-19 patients[7, 12, 13, 15].

**SARS-CoV-2 infected cardiomyocytes recruit monocytes by secreting CCL2.**

Macrophages include tissue-resident macrophages and migrating macrophages[20]. Migrating macrophages are typically derived from monocytes in the blood. During inflammation, circulating monocytes leave the bloodstream and migrate into tissues where, following conditioning by local growth factors, pro-inflammatory cytokines and microbial products, they differentiate into macrophages[1]. CCL2 is a chemotactant for monocytes and basophils. As such, we hypothesized that CCL2 expression of infected CMs attracts monocytes to the site of infection.

To investigate this hypothesis, we therefore examined the ability of SARS-CoV-2 infected CMs to stimulate migration and recruitment of monocytes. Monocytes were derived from the same parental H9 or H1 hESC line following a previously reported protocol [21] (**Extended Data Fig. 2a**) through a stepwise manner, including the generation of mesodermal cells, followed by hematopoietic progenitor cells, monocytes (**Extended Data Fig. 2b**), and finally $CD14^+$, $CD11B^+$ macrophages (**Extended Data Fig. 2c**). To study recruitment, hPSC-derived CMs were plated on the bottom of trans-well plates and hPSC-derived monocytes were plated on top of the insert (**Fig. 4a**). 24 hpi of CMs, the number of migrated monocytes was significantly higher when cultured with SARS-CoV-2 infected hPSC-derived CMs than when cultured with mock infected hPSC-derived CMs using two different hPSC-derive monocytes (**Fig. 4b, 4c** and **Extended Data Fig. 3a, 3b**). These findings were subsequently validated using adult human CMs, also showing that monocytes were recruited at a significantly higher rate when cultured with infected rather than mock infected adult human CMs (**Fig. 4d, 4e** and **Extended Data Fig. 3c, 3d**).

To determine whether CCL2 is sufficient to recruit monocyte, CCL2 was added to the lower level of transwell plates with monocytes embedded in the insert. After 24 h after CCL2 treatment, a significantly higher number of monocytes were found to have migrated to the bottom of the plate compared to mock treated plates (**Fig. 4f, 4g** and **Extended Data Fig. 3e, 3f**). To determine whether CCL2 is the key driver for monocyte migration, hPSC-derived or adult human CMs co-culture assays with monocytes were infected with SARS-CoV-2 in the presence or absence of CCL2 neutralizing antibodies or a CCR2 inhibitor (**Fig. 4h-4k**, and **Extended Data Fig. 3g-3j**). When thereby blocking CCL2 action, significantly less migrating monocytes were detected after viral infection. Together, these data suggest that monocytes are directly recruited to infected CMs by CCL2 secretion.

**Co-culture of hPSC-derived cardiomyocytes and macrophages reveals that macrophages can reduce SARS-CoV-2 infection of CMs.**

We next investigated how recruited macrophages affect the viral infection. To model the viral entry process, we created an immunocardiac co-culture platform containing hPSC-derived CMs and hPSC-derived macrophages. This immunocardiac co-culture was infected with SARS-CoV-2

entry virus carrying a luciferase (Luc) reporter (MOI=0.1) or mock-infected as described previously [10]. At 24 hpi, cells were monitored for Luc activity. The presence of macrophages significantly decreased the Luc activity in a dose-dependent manner (**Extended Data Fig. 4a, 4b**). Immunostaining further confirmed the decrease of $Luc^+$ cells in MYH6:$mCherry^+$ cells (**Extended Data Fig. 4c, 4d**). The immunocardiac co-culture was further examined by scRNA-seq at 24 hpi. The transcript profiling data was projected using Uniform Manifold Approximation and Projection (UMAP). In the virus-immunocardiac co-culture platform (immunocardiac co-culture infected with virus), four distinct cell clusters were identified, including CMs, macrophages, stem/progenitor cells, and one cluster expressing both CM and macrophage markers (**Fig. 5a**). The expression of marker genes, including *MYH6, MYH7, TNNT2* (CMs), *CD163 and CD68* (macrophages), *GATA6* (progenitor cells) in each cell population confirmed the robustness of the cell type classification strategy (**Fig. 5b** and **Extended Data Fig. 4e, 4f**).

The putative viral receptor *ACE2* is expressed mainly in hPSC-derived CMs and cardiac progenitors (**Extended Data Fig. 4g, 4h**). The effector protease *TMPRSS2* [22] is not obviously expressed in hPSC-derived cardiac progenitors (**Extended Data Fig. 4g, 4h**). However, *FURIN,* the gene encoding a pro-protein convertase that pre-activates SARS-CoV-2 [23], and *CTSL,* the gene encoding cathepsin L a proteinase that might be able to substitute for TMPRSS2 [22], are highly expressed in both hPSC-derived CMs and cardiac progenitors (**Extended Data Fig. 4g, 4h**).

The mRNAs derived from SARS-CoV-2 entry virus, including *Luc*, were detected in infected CMs, but at very low levels in macrophages (**Extended Data Fig. 4i, 4j**), which is consistent with our previous report [24]. The one cell cluster that expressed markers of both CMs and macrophages, and in addition high levels of viral genes, likely represents infected CMs engulfed by macrophages (**Fig. 5c**). The *Luc* expression in CMs of virus-immunocardiac co-cultures was much lower than that of virus infected CMs (**Fig. 5d, 5e**), suggesting that macrophages decreased the infection of SARS-CoV-2-pseudo entry virus to CMs. Consistently, the infected CMs show increased expression of CCL2 (**Extended Data Fig. 4k, 4l**).

To further validate the impact of macrophages on SARS-CoV-2 infection, the immunocardiac co-culture platform containing hPSC-derived CMs and hPSC-derived macrophages were infected with SARS-CoV-2 (MOI=0.1) or mock infected. At 24 hpi, cells were analyzed using either qRT-PCR or immunostaining. The qRT-PCR of replicating viral RNA normalized to a cardiomyocyte marker, *cTNT*, suggested significantly decreased SARS-CoV-2 infection (**Fig. 5f**). Immunostaining further validated the decrease of SARS-CoV-$2^+$ in $cTNT^+$ cells (F**ig. 5g, 5h** and **Extended Data Fig. 4m, 4n**). We further performed long-term co-culture of hPSC-derived CMs and macrophages and confirmed that the presence of macrophages decreased SARS-CoV-2 infection to CMs when co-cultured with macrophages for one week (**Fig. 5i, 5j**). Together, the data suggest that macrophages decrease SARS-CoV-2 infection of CMs.

### Discussion

Myocardial injury has been reported in COVID-19 patients and is associated with increased mortality[4, 25], yet the cause of myocardial injury has not been characterized or elucidated. Recent studies using SARS-CoV-2 hACE2 transgenic mice or hPSC-derived CMs reported the detection of SARS-CoV-2 viral RNA in the mouse heart or in SARS-CoV-2 infected CM[26]. In addition, SARS-CoV-2 RNAs have been detected in the heart of COVID-19 autopsy samples by several groups[12, 13]. However, most current data only reported the identification of viral particles in the interstitial cells of the myocardium of COVID-19 patients[7, 13]. The only reported detection of SARS-CoV-2 viral particles in cardiac tissue is a case report of a COVID-19 patient who died after 1 day of admission to hospital [14]. This led to the hypothesis that the failure to detect SARS-CoV-2 viral particles in cardiac tissue in many studies might be because most autopsy samples are collected several weeks after infection. Thus, using an animal model, we examined the hearts of infected hamsters at 2 dpi and clearly detected SARS-N in CMs of SARS-CoV-2 infected hamsters. This provides direct evidence that SARS-CoV-2 infects CMs *in vivo*.

Transcript profiling of SARS-CoV-2 infected hPSC-derived CMs and adult CMs identified significant upregulation of CCL2. CCL2 levels were also significantly upregulated in the hearts of SARS-CoV-2 infected hamsters. CCL2, also known as monocyte chemoattractant protein 1 (MCP-1), is a chemokine that facilitates the migration and infiltration of monocytes/macrophages to sites of inflammation produced by either tissue injury or infection[27]. Using a trans-well platform, we showed that hPSC-derived CMs or adult human CMs infected with SARS-CoV-2 are capable of recruiting migration of monocytes. Consistent with these findings, cell-mixture deconvolution using RNA-seq data identified the enrichment of macrophages in the LA, LV and RA of SARS-CoV-2 infected hamsters. This is consistent with previous reports of abnormal macrophage infiltration in hearts of COVID-19 patients[7, 12, 13, 15].

Finally, we created a co-culture platform using hPSC-derived CMs and macrophages to study the impact of macrophage on CMs. scRNA-seq suggested that the presence of macrophages decreases viral infection. Cell-mixture deconvolution identified the enrichment of pro-inflammatory macrophages in the LA, LV and RA of SARS-CoV-2 infected hamsters. However, RNA-seq analysis of hamster hearts indicated the enhanced production of nitric oxide and ROS in macrophages (**Fig. 1d**), suggesting that macrophages recruited by CMs might also contribute to immune-mediated CM inflammation in COVID-19 patients.

In summary, we provide direct evidence using a hamster model for SARS-CoV-2 infection of CMs *in vivo*. The hearts of SARS-CoV-2 infected hamsters showed downregulation of functional CM associated genes, upregulation of ROS associated genes, and increased CCL2 expression and macrophage infiltration, which was further confirmed using autopsy samples of COVID-19 patients. We developed a trans-well platform containing hPSC-derived CMs and monocytes and found CMs secret increased CCL2 to recruit monocytes upon SARS-CoV-2 infection. Finally, we showed that macrophages could reduce virus infection. This establishes an *in vitro* model to study SARS-CoV-2 infection of cardiac cells and immune-cell infiltration in COVID-19 patients.

### Methods

#### Propagation and titration of SARS-CoV-2

SARS-CoV-2, isolate USA-WA1/2020 (NR-52281) was deposited by the Center for Disease Control and Prevention and obtained through BEI Resources, NIAID, NIH. SARS-CoV-2 was propagated in Vero E6 cells in DMEM supplemented with 2% FBS. Virus stocks were filtered and concentrated by centrifugation using Amicon Ultra-15 Centrifugal filter units (100 KDa MWCO). Infectious titers were determined by plaque assays in Vero E6 cells in Minimum Essential Media supplemented with 2% FBS, 4 mM L-glutamine, 0.2% BSA, 10 mM HEPES and 0.12% $NaHCO_3$ and 0.7% OXOID agar as has been described previously[28].

All work involving live SARS-CoV-2 was performed in the CDC/USDA-approved BSL-3 facility of the Global Health and Emerging Pathogens Institute at the Icahn School of Medicine at Mount Sinai in accordance with institutional biosafety requirements.

#### SARS-CoV-2 infections of Hamsters

3-5-week-old male Golden Syrian hamsters (Mesocricetus auratus) were obtained from Jackson Laboratories. Hamsters were acclimated to the CDC/USDA-approved BSL-3 facility of the Global Health and Emerging Pathogens Institute at the Icahn School of Medicine at Mount Sinai for 2-4 days. Before intranasal infection, hamsters were anesthetized by intraperitoneal injection with a ketamine HCl/xylazine solution (4:1). Hamsters were intranasally inoculated with 100 pfu of SARS-CoV-2 in PBS (or PBS only as a control) in a total volume of 100 µl. Two days post-infection hamsters were euthanized and hearts were collected. For hearts analyzed by immunofluorescence staining, hamsters were perfused with 60 ml of ice-cold PBS before tissue collection and collected hearts were immediately placed in 10% nonbuffered formalin (NFB) and fixed for 24 hours. For transcriptomic analysis, collected hearts were placed in TRIzol for further RNA extraction.

**SARS-CoV-2 live virus infection**

The immunocardiac co-culture containing hPSC-derived CMs and macrophages were infected with SARS-CoV-2 at an MOI of 0.1 and incubated at 37°C for 24 h. Infected cells were either lysed in TRIzol for RNA analysis or fixed in 5% formaldehyde for 24 h for immunofluorescence staining, prior to safe removal from the BSL-3 facility.

**hPSC-derived cardiomyocyte differentiation**

To differentiate cardiomyocytes (CMs) from hPSC, hPSCs were passaged at a density of $3x10^5$cells/well of 6-well plate and grown for 48 hours in a humidified incubator with 5% $CO_2$ at 37°C to reach 90% confluence. On day 0, the medium was replaced with RPMI 1640 supplemented with B27 minnus insulin and 6 μM CHIR99021. On day 1, the medium was changed to RPMI 1640 supplemented with B27 minus insulin for 48 h. Day 3, medium was refreshed to RPMI 1640 supplemented with B27 minus insulin and 2 μM C59 for 48 h. On day 5, the medium was changed back to RPMI-B27 minus insulin for 48 h, and then switched to RPMI 1640 plus normal B27 until day 12. The medium was changed every the other day. On day 12, the medium was transiently changed to RPMI 1640 without D-glucose containing ascorbic acid, human albumin and DL-Lactate for two days to allow metabolic purification of CMs. From that day on, fresh RPMI 1640 plus normal B27 was changed every two days. On day 21, cells were dissociated with Accutase at

37°C followed by resuspending with fresh RPMI 1640-B27 plus Y-27632 and reseeding into new plates. After 24 h, medium was switched to RMPI 1640 plus normal B27 without Y-27632 for following experiments. CMs were derived from two hPSC cell lines: H9-MYH6: Cherry ES cells and WT-F5 iPSC cells. The protocol details are summarized in Extended Data Fig. 1a.

**Adult human cardiomyocytes**

Adult human cardiomyocytes were bought from PromoCell and (Primary Human Cardiac Myocytes, C-12810) cultured in Myocyte Growth Medium (PromoCell, C-22070). Cells were incubated at 37°C with 5% $CO_2$.

**hPSC-derived monocyte and macrophage differentiation**

Monocytes and macrophages were derived from two hPSC lines: H9 ES cells and H1 ES cells. The differentiation protocol was adapted from a previously reported protocol [21]. First, hPSC cells were lifted with ReLeSR (STEMCELL Technologies) as small clusters onto Matrigel-coated 6-well plates at a low density. After 1 day, medium was refreshed with IF9S medium supplemented with 50 ng/ml BMP-4, 15 ng/ml Activin A and 1.5 μm CHIR99021. On day 2, medium was refreshed with IF9S medium supplemented with 50 ng/ml VEGF, 50 ng/ml bFGF, 50 ng/ml SCF (R&D Systems) and 10 μm SB431542 (Cayman Chemical). On day 5 and day7, medium was changed into IF9S supplemented with 50 ng/ml IL-6 (R&D Systems), 12 ng/ml IL-3 (R&D Systems), 50 ng/ml VEGF, 50 ng/ml bFGF, 50 ng/ml SCF and 50 ng/ml TPO (R&D Systems). On day 9, cells were dissociated with TrypLE (Life Technologies) and resuspended in IF9S medium supplemented with 50 ng/ml IL-6, 12 ng/ml IL-3 and 80 ng/ml M-CSF (R&D Systems) into low attachment plates. On day 13 and day 15, medium was changed into IF9S supplemented with 50 ng/ml IL-6, 12 ng/ml IL-3 and 80 ng/ml M-CSF. Monocytes could be collected on day 15. For macrophage differentiation, monocytes were plated onto FBS-coated plates with IF9S medium supplemented with 80ng/ml M-CSF. All differentiation steps were cultured under normoxic conditions at 37 °C, 5% $CO_2$. The protocol details are summarized in Extended Data Fig. 2a.

**Monocyte migration assay**

The migration of macrophages was examined using 24 well Trans-well chambers (6.5 mm insert; 3.0 μm polycarbonate membrane). The upper well was coated with Matrigel before seeding with macrophages ($2X10^4$ cells). After 24 h, the chamber was fixed and stained with crystal violet. Migrated cells were counted under an inverted light microscope.

**The immunocardiac co-culture**

hPSC-derived cardiomyocytes were dissociated with Accutase for 5-10min at 37°C followed by resuspending with fresh RPMI 1640 plus normal B27 and Y-27632 and reseeding into plates. After 24 h recovery, the medium was switched to RMPI 1640 plus B27 without Y-27632. After another 24 h recovery, hPSC-derived macrophages were dissociated with Accutase for 3 min and added into hPSC-derived cardiomyocytes. The immunocardiac co-culture cells were cultured for another 24 h (short-term co-culture) or 7 days (long-term co-culture) before following analysis. Adult cardiomyocytes were also seeded into plates for 48-96 h and co-cultured with hPSC-derived macrophages for another 24 h before following analysis.

### Cell Lines

HEK293T (human [*Homo sapiens*] fetal kidney) and Vero E6 (African green monkey [*Chlorocebus aethiops*] kidney) were obtained from ATCC (https://www.atcc.org/). Cells were cultured in Dulbecco's Modified Eagle Medium (DMEM) supplemented with 10% FBS and 100 I.U./mL penicillin and 100 µg/mL streptomycin. All cell lines were incubated at 37°C with 5% $CO_2$.

### SARS-CoV-2 entry virus infection

To assay entry-typed virus infection, cells were seeded into 96 well plates. Pseudo-typed virus was added at the indicated MOI.  At 2 hpi, the infection medium was replaced with fresh medium. At 24 hpi, cells were harvested for luciferase assay following the Luciferase Assay System protocol (E1501, Promega) or immunostaining analysis.

### Immunostaining

Hamster heart tissues were obtained from mock or SARS-CoV-2 infected hamsters. Heart tissues were fixed overnight in 5% formaldehyde, sink in 30% surcose and embed in OCT (Fisher Scientific, Pittsburgh, PA). The slices were wash with PBS 2 times to remove OCT and incubated in 0.1% Triton for 1h at room temperature. Then, slices were stained with primary antibodies at 4°C overnight and secondary antibodies at RT for 1h. The information for primary antibodies and secondary antibodies is provided in Extended Data Table 1. Nuclei were counterstained by DAPI.

### qRT-PCR

Total RNA samples were prepared from tissues or cells using TRIzol and Direct-zol RNA Miniprep Plus kit (Zymo Research) according to the manufacturer's instructions. To quantify viral replication, measured by the expression of sgRNA transcription of the viral N gene, one-step quantitative real-time PCR was performed using SuperScript III Platinum SYBR Green One-Step qRT-PCR Kit (Invitrogen) with primers specific for the TRS-L and TRS-B sites of the *N* gene as well as *ACTB* or *cTNT* as an internal reference. Quantitative real-time PCR reactions were performed on a LightCycler 480 Instrument II (Roche). Delta-delta-cycle threshold (ΔΔCT) was determined relative to the *ACTB* or *cTNT* and mock infected /treated samples. Error bars indicate the standard deviation of the mean from three biological replicates. The sequences of primers/probes are provided in Extended Data Table 2.

### ELISA

CCL2 levels in the supernatant of infected hPSC-derived CMs were evaluated using ELISA. The antibody and cytokine standards were purchased as antibody pairs from R&D Systems (Minneapolis, Minnesota) or Peprotech (Rocky Hill, New Jersey). Individual magnetic Luminex bead sets (Luminex Corp, CA) were coupled to cytokine-specific capture antibodies according to the manufacturer's recommendations. The assays were read on a MAGPIX platform. The median fluorescence intensity of these beads was recorded for each bead and was used for analysis using a custom R script and a 5P regression algorithm.

### Sequencing and gene expression UMI counts matrix generation

The 10X libraries were sequenced on the Illumina NovaSeq6000 sequencer with pair-end reads (28 bp for read 1 and 91 bp for read 2). The sequencing data were primarily analyzed by the 10X cellranger pipeline (v3.0.2) in two steps. In the first step, cellranger *mkfastq* demultiplexed samples and generated fastq files; and in the second step, cellranger count aligned fastq files to the reference genome and extracted gene expression UMI counts matrix. In order to measure viral gene expression, we built a custom reference genome by integrating the four virus genes, luciferase and two fluoresence genes into the 10X pre-built human reference (GRCh38 v3.0.0) using cellranger *mkref*. The sequences of four viral genes (VSV-N, VSV-NS, VSV-M and VSV-L) were retrieved from NCBI (https://www.ncbi.nlm.nih.gov/nuccore/335873), the sequence of the luciferase was retrieved from HIV-Luc, and the sequences of the two fluorescence genes were downloaded from NCBI (mCherry: https://www.ncbi.nlm.nih.gov/nuccore/AY678264.1; GFP: https://www.ncbi.nlm.nih.gov/nuccore/U55761.1).

### Single-cell RNA-seq data analysis

We filtered a small fraction of cells with viral gene content greater than 80% but less than 200 genes detected for which we believe are not real cells but rather empty beads with ambient RNAs. We then filtered cells with less than 400 or more than 7000 genes detected as well as cells with mitochondria gene content greater than 15%, and used the remaining cells (1654 cells for CM; 1555 cells for CM+virus; 4001 cells for CM+macrophage+virus) for downstream analysis. We normalized the gene expression UMI counts using a deconvolution strategy implemented by the R scran package (v.1.14.1). In particular, we pre-clustered cells using the *quickCluster* function; we computed size factor per cell within each cluster and rescaled the size factors by normalization between clusters using the *computeSumFactors* function; and we normalized the UMI counts per cell by the size factors and took a logarithm transform using the *normalize* function. We further normalized the UMI counts across samples using the *multiBatchNorm* function in the R batchelor package (v1.2.1). We identified highly variable genes using the *FindVariableFeatures* function in the R Seurat package (v3.1.0) [29], and selected the top 3000 variable genes after excluding mitochondria genes, ribosomal genes, dissociation-related genes, viral genes and fluorescence genes. The list of dissociation-related genes was originally built on mouse data [30]; we converted them to human ortholog genes using Ensembl BioMart. We aligned the two samples based on their mutual nearest neighbors (MNNs) using the *fastMNN* function in the R batchelor package, this was done by performing a principal component analysis (PCA) on the highly variable genes and then correcting the principal components (PCs) according to their MNNs. We selected the corrected top 50 PCs for downstream visualization and clustering analysis. We ran UMAP dimensional reduction using the *RunUMAP* function in the R Seurat package with the number of neighboring points setting to 35 and training epochs setting to 2000. We clustered cells into fifteen clusters by constructing a shared nearest neighbor graph and then grouping cells of similar transcriptome profiles using the *FindNeighbors* function and *FindClusters* function (resolution set to 0.7) in the R Seurat package. We identified marker genes for each cluster by performing differential expression analysis between cells inside and outside that cluster using the *FindMarkers* function in the R Seurat package. After reviewing the clusters, we merged them into four clusters representing macrophages, CM, CM+macrophages and progenitor cells, for further analysis. We re-identified marker genes for the merged four clusters and selected top 10 positive marker genes per cluster for heatmap plot using the *DoHeatmap* function in the R Seurat package. The rest plots were generated using the R ggplot2 package.

### RNA-Seq before and following viral infections

RNAseq libraries of polyadenylated RNA were prepared using TruSeq Stranded mRNA Library Prep Kit (Illumina) according to the manufacturer's instructions. cDNA libraries were sequenced using an Illumina NextSeq 500 platform. The sequencing reads were cleaned by trimming adapter sequences and low quality bases using cutadapt v1.9.1 [31], and were aligned to the human reference genome (GRCh37) or the SARS-CoV-2 genome (NC_045512.2) using STAR v2.5.2b [32]. Raw gene counts were quantified using HTSeq-count v0.11.2 [33]. Differential expression analysis was performed using DESeq2 v1.22.2 [34]. Regularized log transformation was applied to convert count data to log2 scale. Sample-to-sample distance matrix was calculated based on the transformed log-scaled count data using R *dist* function. Multidimensional scaling (MDS) was performed on the distance matrix using R *cmdscale* function.  The RNA sequencing reads of hamster heart samples were aligned to hamster reference genome (download from Ensembl, accession#: GCA 000349665) plus SARS-Cov-2 genome using HISAT2 2.1.0. Raw gene counts were quantified using HTSeq-count v0.11.2. PCA plot was drawed using R functions prcomp.

### Intracellular flow cytometry analysis

Flow cytometry staining was performed to examine the expression of CD14 and CD11B. Briefly, Cells were dissociated with Acctuase, then wash twice with PBS containing 0.5% BSA and 2mM EDTA. Incubate with antibody at 4°C for 1 h in the dark, wash twice and then do flow cytometry analysis. The information for primary antibodies and secondary antibodies are provided in Extended Data Table 1.

**Human studies**

For RNA analysis, tissue was acquired from deceased COVID19 human subjects during autopsy and processed in TRIZOL. Tissue samples were provided by the Weill Cornell Medicine Department of Pathology. The uninfected human heart samples were similarly obtained. The Tissue Procurement Facility operates under Institutional Review Board (IRB) approved protocol and follows guidelines set by HIPAA. Experiments using samples from human subjects were conducted in accordance with local regulations and with the approval of the institutional review board at the Weill Cornell Medicine under protocol 20-04021814.

**Quantification and Statistical analysis**

N=3 independent biological replicates were used for all experiments unless otherwise indicated. n.s. indicates a non-significant difference. *P*-values were calculated by unpaired two-tailed Student's t-test unless otherwise indicated. $*p<0.05$, $**p<0.01$ and $***p<0.001$.

**Data and Code Availability**

scRNA-seq and RNA-seq data are available from the GEO repository database with accession number GSE151880. (Reviewer Token: ctgpukaevxkdtwh).

### Author contributions

S. C., T. E., B. T., R.E.S., and D. D. H., conceived and designed the experiments. L. Y., Y.H., F.J., and J. Z., performed CM, macrophage differentiation, co-culture, and immunostaining. J.A.A, J.K.L, performed ELISA analysis. P. W, Y. H., provided SARS2-CoV-2 pseudo-entry virus. A.B., Y.B., C.R, V.C, analyzed human samples. B. N., R.M., D.A.H, S.H., and B. T., performed SARS2-CoV-2 related experiments. J.Z., T. Z., D. R., S. H., J. X. Z., performed the scRNA-sequencing and bioinformatics analyses.

### Acknowledgments

This work was supported by the American Heart Association (18CSA34080171, S.C., T.E.), Department of Surgery, Weill Cornell Medicine (T.E., S.C.), and (NCI R01CA234614, NIAID 2R01AI107301 and NIDDK R01DK121072 and 1RO3DK117252), Department of Medicine, Weill Cornell Medicine (R.E.S.), by the Defense Advanced Research Projects Agency (DARPA-16-35-INTERCEPT-FP-006, B.T.) and by the Jack Ma Foundation (D.D.H). S.C and R.E.S. are supported as Irma Hirschl Trust Research Award Scholars.

**Conflict of interest.** R.E.S. is on the scientific advisory board of Miromatrix Inc. The other authors have no conflict of interest.

## Figure 1

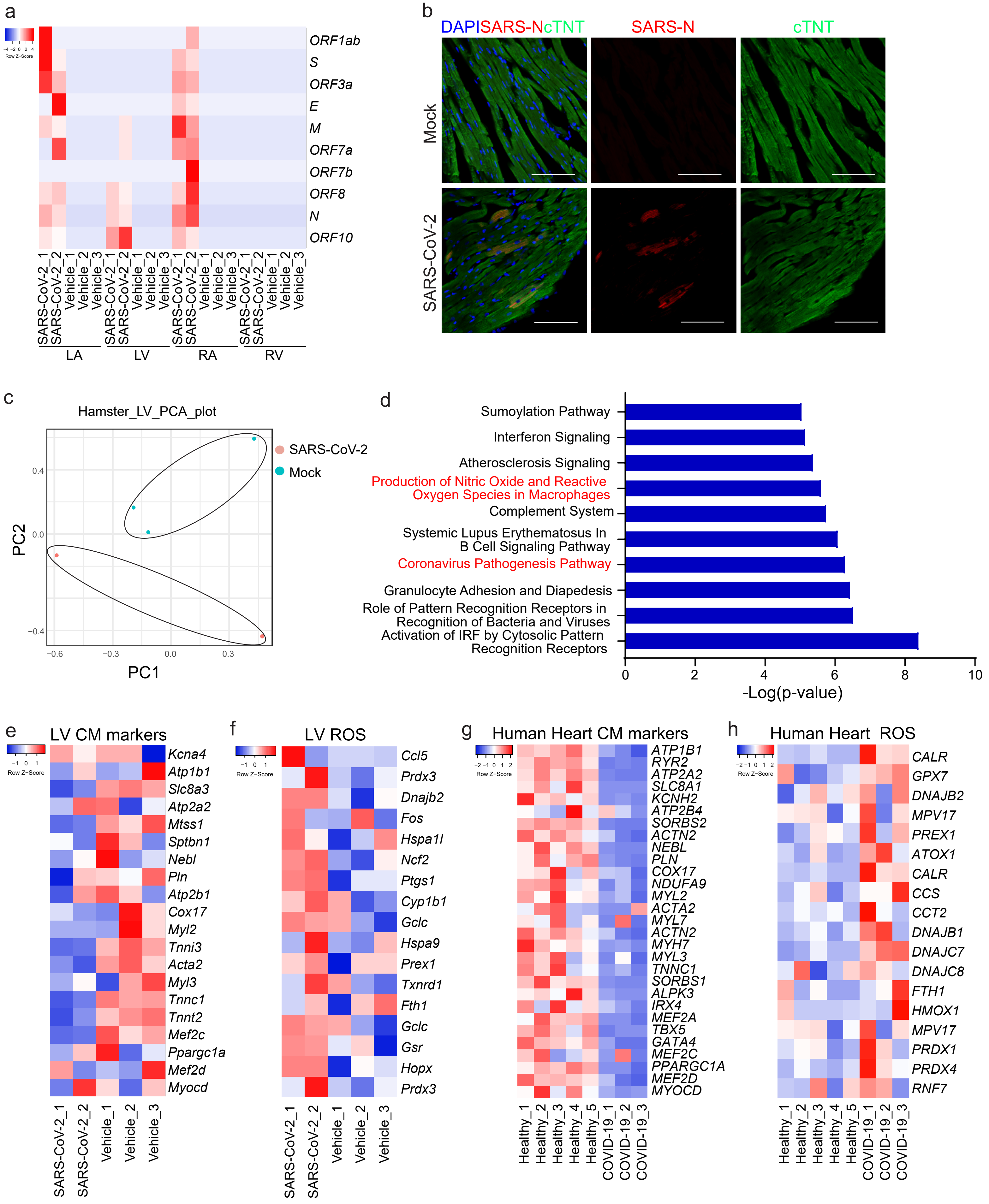

a
Row Z-Score
ORF1ab
S
ORF3a
E
M
ORF7a
ORF7b
ORF8
N
ORF10
SARS-CoV-2_1
SARS-CoV-2_2
Vehicle_1
Vehicle_2
Vehicle_3
LA
LV
RA
RV
b
DAPI SARS-N cTNT
SARS-N
cTNT
Mock
SARS-CoV-2
c
Hamster_LV_PCA_plot
SARS-CoV-2
Mock
PC2
PC1
0.4
0.0
−0.4
−0.6
−0.3
0.0
0.3
d
Sumoylation Pathway
Interferon Signaling
Atherosclerosis Signaling
Production of Nitric Oxide and Reactive Oxygen Species in Macrophages
Complement System
Systemic Lupus Erythematosus In B Cell Signaling Pathway
Coronavirus Pathogenesis Pathway
Granulocyte Adhesion and Diapedesis
Role of Pattern Recognition Receptors in Recognition of Bacteria and Viruses
Activation of IRF by Cytosolic Pattern Recognition Receptors
0
2
4
6
8
10
-Log(p-value)
e
LV CM markers
Row Z-Score
Kcna4
Atp1b1
Slc8a3
Atp2a2
Mtss1
Sptbn1
Nebl
Pln
Atp2b1
Cox17
Myl2
Tnni3
Acta2
Myl3
Tnnc1
Tnnt2
Mef2c
Ppargc1a
Mef2d
Myocd
SARS-CoV-2_1
SARS-CoV-2_2
Vehicle_1
Vehicle_2
Vehicle_3
f
LV ROS
Row Z-Score
Ccl5
Prdx3
Dnajb2
Fos
Hspa1l
Ncf2
Ptgs1
Cyp1b1
Gclc
Hspa9
Prex1
Txnrd1
Fth1
Gclc
Gsr
Hopx
Prdx3
SARS-CoV-2_1
SARS-CoV-2_2
Vehicle_1
Vehicle_2
Vehicle_3
g
Human Heart CM markers
Row Z-Score
ATP1B1
RYR2
ATP2A2
SLC8A1
KCNH2
ATP2B4
SORBS2
ACTN2
NEBL
PLN
COX17
NDUFA9
MYL2
ACTA2
MYL7
ACTN2
MYH7
MYL3
TNNC1
SORBS1
ALPK3
IRX4
MEF2A
TBX5
GATA4
MEF2C
PPARGC1A
MEF2D
MYOCD
Healthy_1
Healthy_2
Healthy_3
Healthy_4
Healthy_5
COVID-19_1
COVID-19_2
COVID-19_3
h
Human Heart ROS
Row Z-Score
CALR
GPX7
DNAJB2
MPV17
PREX1
ATOX1
CALR
CCS
CCT2
DNAJB1
DNAJC7
DNAJC8
FTH1
HMOX1
MPV17
PRDX1
PRDX4
RNF7
Healthy_1
Healthy_2
Healthy_3
Healthy_4
Healthy_5
COVID-19_1
COVID-19_2
COVID-19_3

### FIGURE LEGENDS

**Figure 1. SARS-CoV-2 is detected in the CMs of SARS-CoV-2 infected hamsters. a,** Heatmap of SARS-CoV-2 viral genes in heart tissues obtained from SARS-CoV-2 infected (N=2) or mock infected (N=3) hamsters. Data was presented as the Z score. **b,** Immunohistochemistry staining of SARS-N in the LV heart tissues obtained from SARS-CoV-2 infected (N=2) or mock infected (N=3) hamsters. Scale bar= 50 μm. **c,** PCA plot of the LV heart tissues obtained from SARS-CoV-2 infected (N=2) or mock infected (N=3) hamsters. **d,** Ingenuity Pathway Analysis (IPA) of pathways enriched in SARS-CoV-2 infected LV heart tissues compared to mock infected LV heart tissues. **e,** Heatmap of CM function associated genes in the LV heart tissues obtained from SARS-CoV-2 infected (N=2) or mock infected (N=3) hamsters. Data was presented as the Z-score. **f,** Heatmap of ROS associated genes in the LV heart tissues obtained from SARS-CoV-2 infected (N=2) or mock infected (N=3) hamsters. Data was presented as the Z-score. **g,** Heatmap of CM function associated genes in autopsy heart samples of healthy donors and COVID-19 patients (N=5 healthy patients, N=3 COVID-19 patients). Data was presented as the Z-score. **h,** Heatmap of ROS associated genes in autopsy heart samples of healthy donors and COVID-19 patients (N=5 healthy patients, N=3 COVID-19 patients). Data was presented as the Z-score. Data was presented as mean ± STDEV. *P* values were calculated by unpaired two-tailed Student's t test. $*P < 0.05$ and $***P < 0.001$.

Figure 2

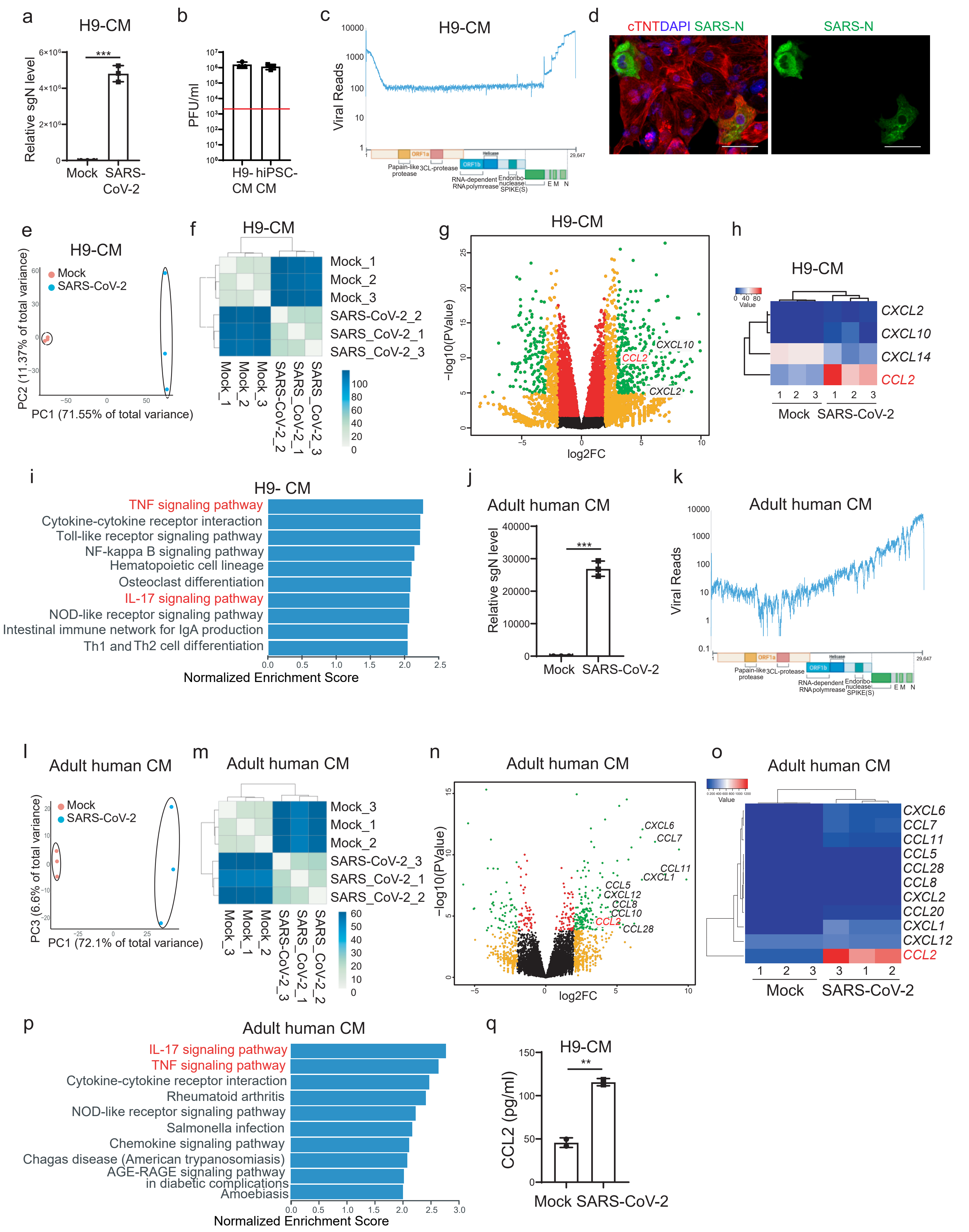

a
H9-CM
Relative sgN level
***
Mock
SARS-CoV-2
b
PFU/ml
H9-CM
hiPSC-CM
c
H9-CM
Viral Reads
ORF1a
Papain-like protease
3CL-protease
ORF1b
Helicase
RNA-dependent RNA polymerase
Endoribo-nuclease
SPIKE(S)
E M N
29,647
d
cTNT DAPI SARS-N
SARS-N
e
H9-CM
Mock
SARS-CoV-2
PC2 (11.37% of total variance)
PC1 (71.55% of total variance)
f
H9-CM
Mock_1
Mock_2
Mock_3
SARS-CoV-2_2
SARS_CoV-2_1
SARS_CoV-2_3
g
H9-CM
−log10(PValue)
log2FC
CXCL10
CCL2
CXCL2
h
H9-CM
Value
CXCL2
CXCL10
CXCL14
CCL2
Mock
SARS-CoV-2
i
H9- CM
TNF signaling pathway
Cytokine-cytokine receptor interaction
Toll-like receptor signaling pathway
NF-kappa B signaling pathway
Hematopoietic cell lineage
Osteoclast differentiation
IL-17 signaling pathway
NOD-like receptor signaling pathway
Intestinal immune network for IgA production
Th1 and Th2 cell differentiation
Normalized Enrichment Score
j
Adult human CM
Relative sgN level
***
Mock
SARS-CoV-2
k
Adult human CM
Viral Reads
l
Adult human CM
Mock
SARS-CoV-2
PC3 (6.6% of total variance)
PC1 (72.1% of total variance)
m
Adult human CM
Mock_3
Mock_1
Mock_2
SARS-CoV-2_3
SARS_CoV-2_1
SARS_CoV-2_2
n
Adult human CM
−log10(PValue)
log2FC
CXCL6
CCL7
CCL11
CXCL1
CCL5
CXCL12
CCL8
CCL10
CCL2
CCL28
o
Adult human CM
Value
CXCL6
CCL7
CCL11
CCL5
CCL28
CCL8
CXCL2
CCL20
CXCL1
CXCL12
CCL2
Mock
SARS-CoV-2
p
Adult human CM
IL-17 signaling pathway
TNF signaling pathway
Cytokine-cytokine receptor interaction
Rheumatoid arthritis
NOD-like receptor signaling pathway
Salmonella infection
Chemokine signaling pathway
Chagas disease (American trypanosomiasis)
AGE-RAGE signaling pathway in diabetic complications
Amoebiasis
Normalized Enrichment Score
q
H9-CM
CCL2 (pg/ml)
**
Mock
SARS-CoV-2

**Figure 2. CMs secret CCL2 upon SARS-CoV-2 infection. a,** Relative viral RNA expression in H9-derived CMs at 24 hpi of SARS-CoV-2 virus (MOI=0.1). **b,** Plaque assay of H9-derived CMs at 24 hpi of SARS-CoV-2 virus (MOI=0.1). **c,** Alignment of the transcriptome with the viral genome in SARS-CoV-2 infected H9-derived CMs. Schematic denotes the SARS-CoV-2 genome. **d,** Immunostaining of cTNT and SARS-N in H9-derived CMs infected with SARS-CoV-2 virus (MOI=0.1) or mock. Scale bar= 50 μm. **e, f,** PCA plot (**e**) and heatmap (**f**) analysis of H9-derived CMs infected with SARS-CoV-2 virus or mock. **g, h,** Volcano plot (**g**) and heatmap (**h**) analysis of chemokines expressed by H9-derived CMs infected with SARS-CoV-2 virus or mock. Colored dots correspond to chemokines with significant ($p<0.05$) and greater than 2-fold expression level changes. **i,** KEGG analysis of H9-derived CMs infected with SARS-CoV-2 virus or mock. **j,** Relative viral RNA expression in adult human CMs at 24 hpi of SARS-CoV-2 virus (MOI=0.1). **k,** Alignment of the transcriptome with the viral genome in SARS-CoV-2 infected adult human CMs. Schematic denotes the SARS-CoV-2 genome. **l, m,** PCA plot (**l**) and heatmap (**m**) analysis of adult human CMs infected with SARS-CoV-2 virus or mock. **n, o,** Volcano plot (**n**) and heatmap (**o**) analysis of chemokines expressed by adult human CMs infected with SARS-CoV-2 virus or mock. Colored dots correspond to chemokines with significant ($p<0.05$) and greater than 2-fold expression level changes. **p,** KEGG analysis of adult human CMs infected with SARS-CoV-2 virus or mock. **q,** ELISA assay was performed to examine the protein level of CCL2 in H9-derived CMs infected with SARS-CoV-2 virus or mock (MOI=0.1). N=3 independent biological replicates. Data was presented as mean ± STDEV. *P* values were calculated by unpaired two-tailed Student's t test. $^{**}P < 0.01$, and $^{***}P < 0.001$.

### Figure 3

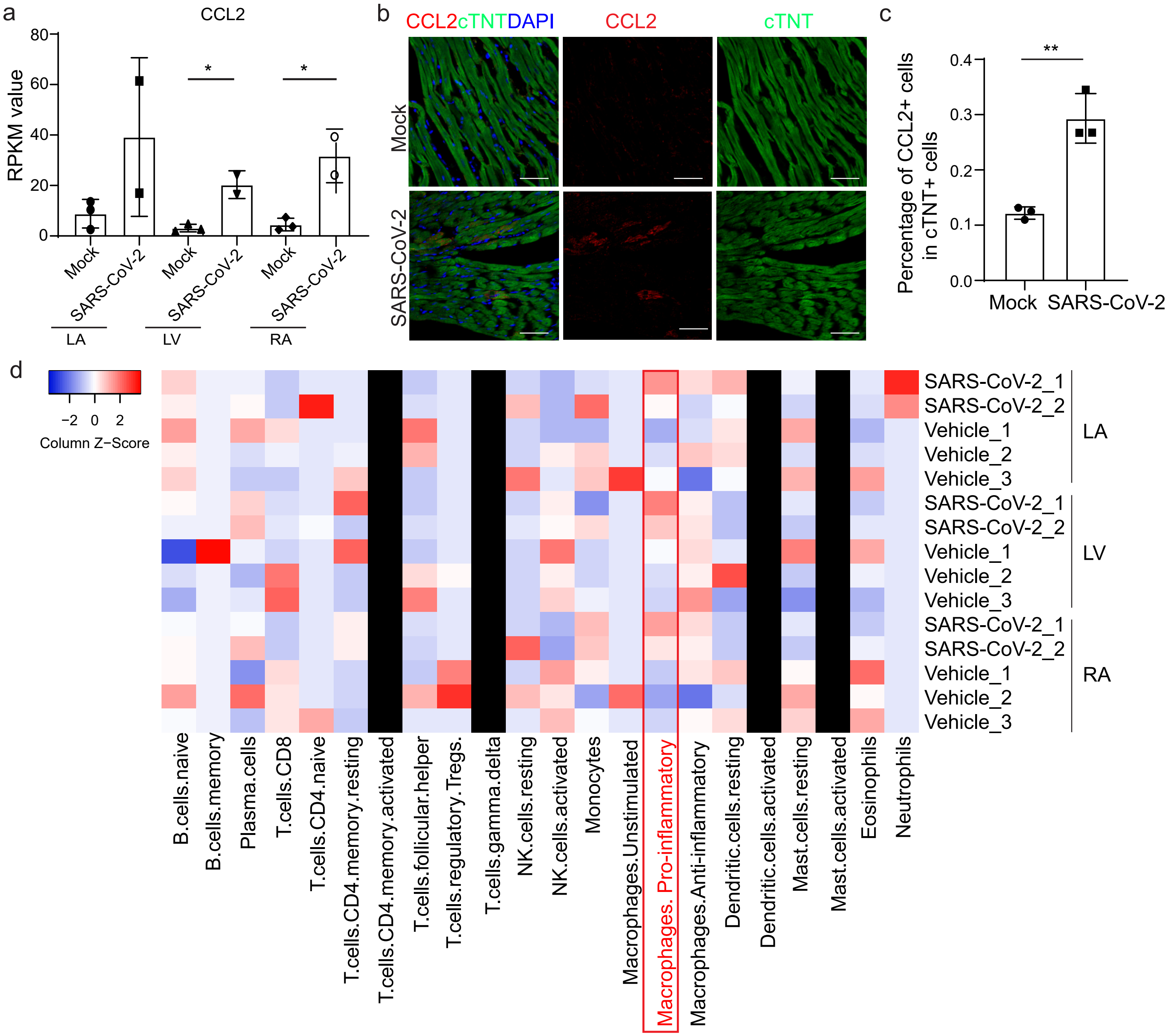

a
CCL2
RPKM value
80
60
40
20
0
*
*
Mock
SARS-CoV-2
Mock
SARS-CoV-2
Mock
SARS-CoV-2
LA
LV
RA
b
CCL2cTNTDAPI
CCL2
cTNT
Mock
SARS-CoV-2
c
Percentage of CCL2+ cells in cTNT+ cells
0.4
0.3
0.2
0.1
0.0
**
Mock
SARS-CoV-2
d
−2 0 2
Column Z−Score
SARS-CoV-2_1
SARS-CoV-2_2
Vehicle_1
Vehicle_2
Vehicle_3
LA
SARS-CoV-2_1
SARS-CoV-2_2
Vehicle_1
Vehicle_2
Vehicle_3
LV
SARS-CoV-2_1
SARS-CoV-2_2
Vehicle_1
Vehicle_2
Vehicle_3
RA
B.cells.naive
B.cells.memory
Plasma.cells
T.cells.CD8
T.cells.CD4.naive
T.cells.CD4.memory.resting
T.cells.CD4.memory.activated
T.cells.follicular.helper
T.cells.regulatory.Tregs.
T.cells.gamma.delta
NK.cells.resting
NK.cells.activated
Monocytes
Macrophages.Unstimulated
Macrophages. Pro-inflammatory
Macrophages.Anti-inflammatory
Dendritic.cells.resting
Dendritic.cells.activated
Mast.cells.resting
Mast.cells.activated
Eosinophils
Neutrophils

**Figure 3. Pro-inflammatory macrophages were enriched in heart of SARS-CoV-2 infected hamsters. a,** RPKM values of *CCL2* in heart tissues obtained from SARS-CoV-2 infected hamsters (N=2) and mock infected hamsters (N=3). **b, c,** Immunohistochemistry staining (b) and quantification (c) of CCL2 in SARS-CoV-2 infected hamsters (N=2) and mock infected hamsters (N=3). Scale bar= 50 μm. **d,** Cell-mixture deconvolution identified the enrichment of immune cells in the LA, LV and RA of SARS-CoV-2 infected (N=2) or mock infected hamsters (N=3). *P* values were calculated by unpaired two-tailed Student's t test. *$P < 0.05$ and **$P < 0.01$.

## Figure 4

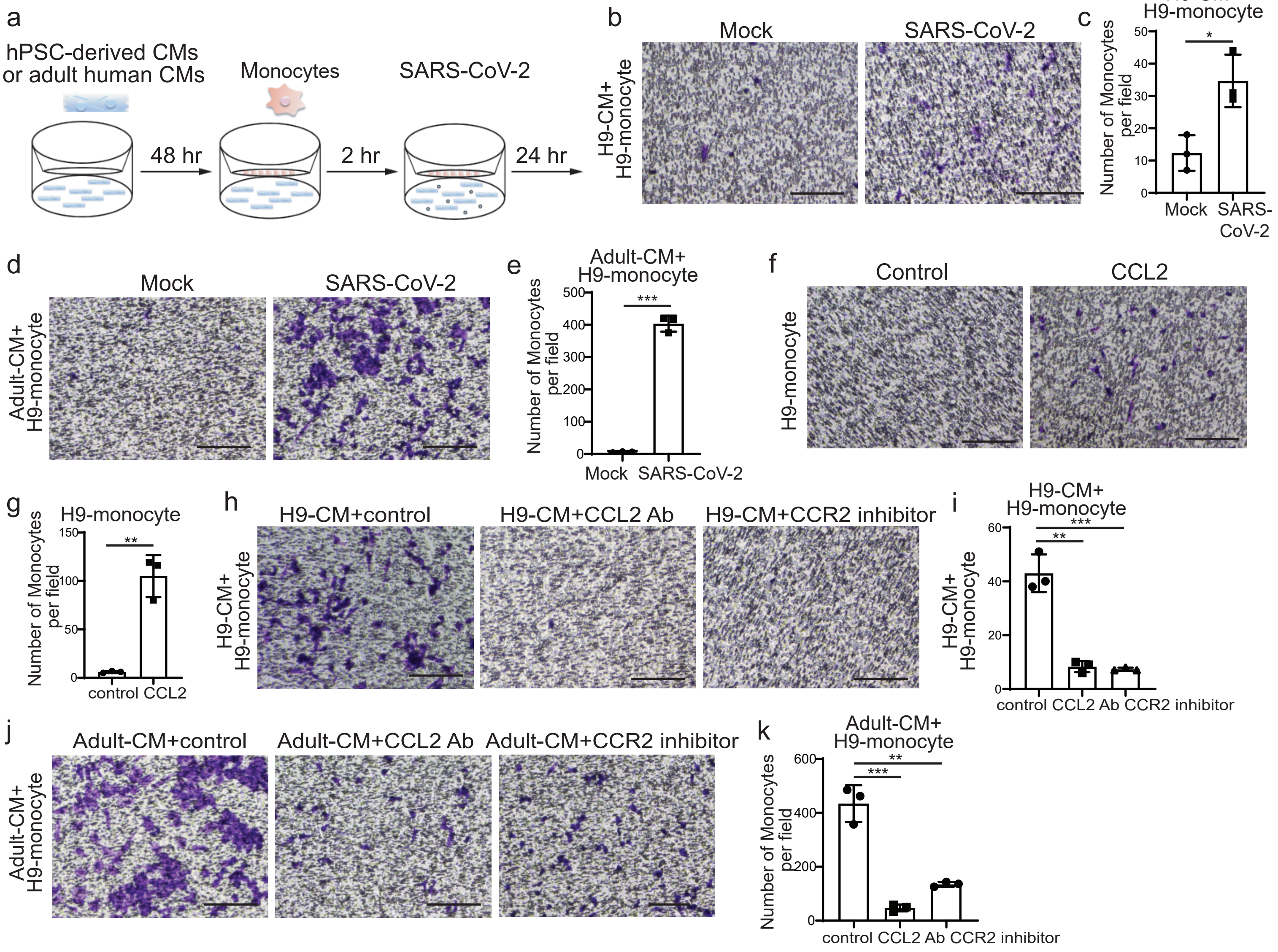

a
hPSC-derived CMs
or adult human CMs
Monocytes
SARS-CoV-2
48 hr
2 hr
24 hr
b
Mock
SARS-CoV-2
H9-CM+
H9-monocyte
c
H9-CM+
H9-monocyte
Number of Monocytes per field
Mock
SARS-CoV-2
d
Mock
SARS-CoV-2
Adult-CM+
H9-monocyte
e
Adult-CM+
H9-monocyte
Number of Monocytes per field
Mock
SARS-CoV-2
f
Control
CCL2
H9-monocyte
g
H9-monocyte
Number of Monocytes per field
control
CCL2
h
H9-CM+control
H9-CM+CCL2 Ab
H9-CM+CCR2 inhibitor
H9-CM+
H9-monocyte
i
H9-CM+
H9-monocyte
H9-CM+
H9-monocyte
control
CCL2 Ab
CCR2 inhibitor
j
Adult-CM+control
Adult-CM+CCL2 Ab
Adult-CM+CCR2 inhibitor
Adult-CM+
H9-monocyte
k
Adult-CM+
H9-monocyte
Number of Monocytes per field
control
CCL2 Ab
CCR2 inhibitor

**Figure 4. CMs recruit monocytes following SARS-CoV-2 infection through secreting CCL2.** **a,** Scheme of the monocyte recruitment assay using hPSC-derived CMs or adult human CMs and hPSC-derived monocytes in the presence of SARS-CoV-2 infection. **b, c,** Phase contract images (b) and quantification (c) of migrated H9-derived monocytes recruited by H9-derived CMs infected with SARS-CoV-2 virus or mock in the monocyte migration assay as described in (a). Scale bar= 100 µm. **d, e,** Phase contract images (d) and quantification (e) of H9-derived monocytes recruited by adult human CMs infected with SARS-CoV-2 virus or mock in the monocyte recruitment assay as described in (a). Scale bar= 100 µm. **f, g,** Phase contrast images (**f**) and quantification (**g**) of migrated H9-derived monocytes by CCL2 in monocyte recruitment assay. Scale bar= 100 µm. **h, i,** Phase contrast images (**h**) and quantification (**i**) of migrated H9-derived monocytes recruited by H9-derived CMs infected with SARS-CoV-2 virus and treated with CCL2 neutralizing antibody or CCR2 inhibitor: RS504393 in monocyte recruitment assay. Scale bar= 100 µm. **j, k,** Phase contrast images (**j**) and quantification (**k**) of migrated H9-derived monocytes recruited by adult human CMs infected with SARS-CoV-2 virus and treated with CCL2 neutralizing antibody or CCR2 inhibitor: RS504393 in monocyte recruitment assay. Scale bar= 100 µm. N=3 independent biological replicates. Data was presented as mean ± STDEV. *P* values were calculated by unpaired two-tailed Student's t test. *$P < 0.05$, **$P < 0.01$, and ***$P < 0.001$.

## Figure 5

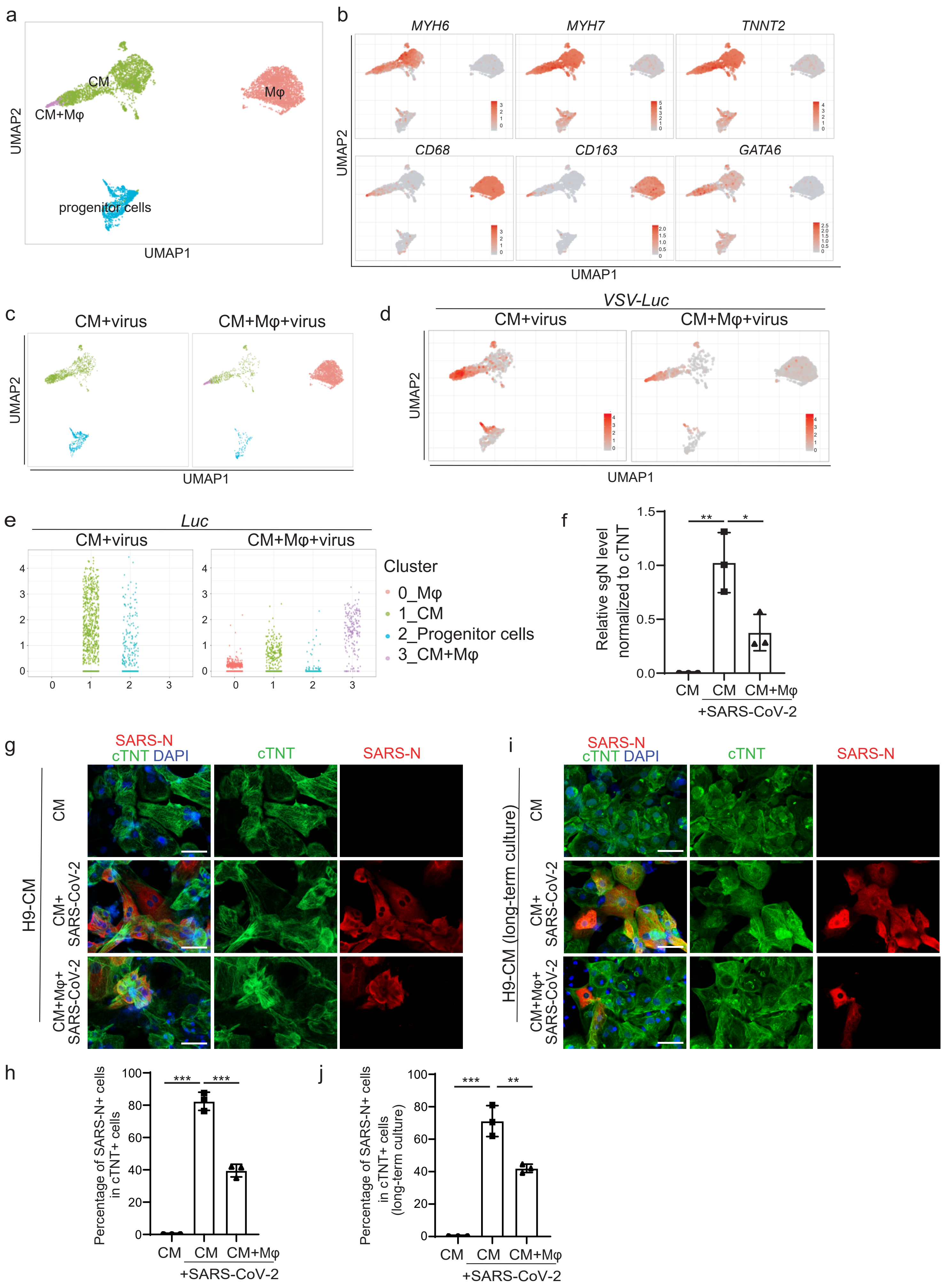

a
CM
Mφ
CM+Mφ
progenitor cells
UMAP1
UMAP2
b
MYH6
MYH7
TNNT2
CD68
CD163
GATA6
UMAP1
UMAP2
c
CM+virus
CM+Mφ+virus
UMAP1
UMAP2
d
VSV-Luc
CM+virus
CM+Mφ+virus
UMAP1
UMAP2
e
Luc
CM+virus
CM+Mφ+virus
Cluster
0_Mφ
1_CM
2_Progenitor cells
3_CM+Mφ
f
Relative sgN level normalized to cTNT
CM
CM
CM+Mφ
+SARS-CoV-2
g
SARS-N
cTNT DAPI
cTNT
SARS-N
H9-CM
CM
CM+ SARS-CoV-2
CM+Mφ+ SARS-CoV-2
i
SARS-N
cTNT DAPI
cTNT
SARS-N
H9-CM (long-term culture)
CM
CM+ SARS-CoV-2
CM+Mφ+ SARS-CoV-2
h
Percentage of SARS-N+ cells in cTNT+ cells
CM
CM
CM+Mφ
+SARS-CoV-2
j
Percentage of SARS-N+ cells in cTNT+ cells (long-term culture)
CM
CM
CM+Mφ
+SARS-CoV-2

**Figure 5. A virus-immunocardiac co-culture platform reveals that hPSC-derived macrophages reduce SARS-CoV-2 infection to hPSC-derived CMs. a,** UMAP analysis of the virus-immunocardiac tissue platform containing hPSC-derived CMs and macrophages, and was infected with SARS-CoV-2-entry virus (MOI=0.1). **b,** UMAP of hPSC-derived CM and macrophage related markers differentially expressed in each cluster. Relative expression levels of each marker gene ranged from low (gray) to high (red) as indicated. **c,** UMAP analysis of clusters in hPSC-derived CMs infected with SARS-CoV-2-entry virus (CM+virus) and the virus-immunocardaic tissue platform containing hPSC-derived CMs and macrophages infected with SARS-CoV-2-entry virus (CM+macrophage+virus). **d,** UMAP analysis of *Luc* expression in hPSC-derived CMs infected with SARS-CoV-2-entry virus (CM+virus) and the virus-immunocardaic tissue platform containing hPSC-derived CMs and macrophages infected with SARS-CoV-2-entry virus (CM+macrophage+virus). **e,** Jitter plot of *Luc* expression in hPSC-derived CMs infected with SARS-CoV-2 entry virus (CM+virus) and the virus-immunocardaic tissue platform containing hPSC-derived CMs and macrophages and infected with SARS-CoV-2-entry virus (CM+macrophage+virus). **f,** qRT-PCR analysis at 24 hpi of hPSC-derived CMs infected with mock or SARS-CoV-2 in the presence or absence of macrophages (MOI=0.1). **g, h,** Immunostaining (**g**) and quantification (**h**) of hPSC-derived CMs at 24 hpi with mock or SARS-CoV-2 in the presence or absence of macrophages (MOI=0.1) for short-time co-culture (24 h). Immunostaining (**i**) and quantification (**j**) of hPSC-derived CMs at 24 hpi with mock or SARS-CoV-2 in the presence or absence of macrophages (MOI=0.1) for long-time co-culture (7 days). N=3 independent biological replicates. Data was presented as mean ± STDEV. *P* values were calculated by unpaired two-tailed Student's t test. **P* < 0.05, ***P* < 0.01 and ****P* < 0.001.

### Extended Data Figure 1

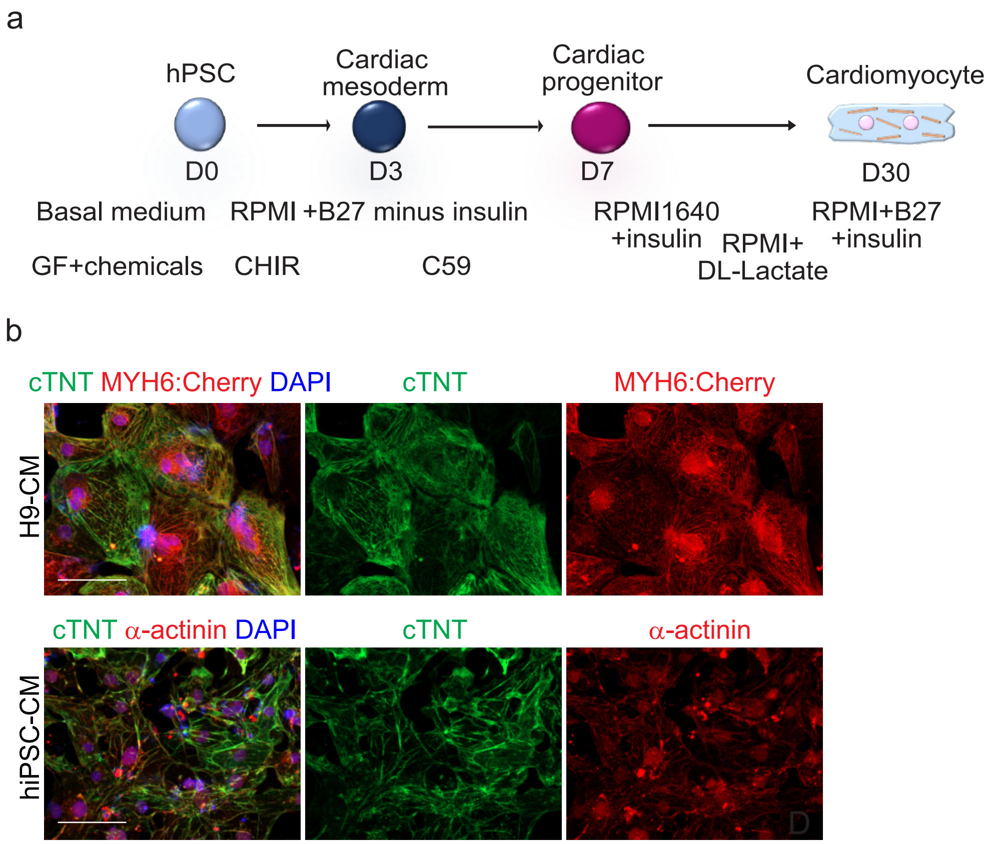

**Extended Data Figure 1. Stepwise differentiation of hPSCs toward CMs. a,** Scheme of stepwise differentiation of hPSCs toward CMs. **b,** Immunostaining of the hPSC-derived CMs. Scale bar= 100 μm.

**Extended Data Figure 2**

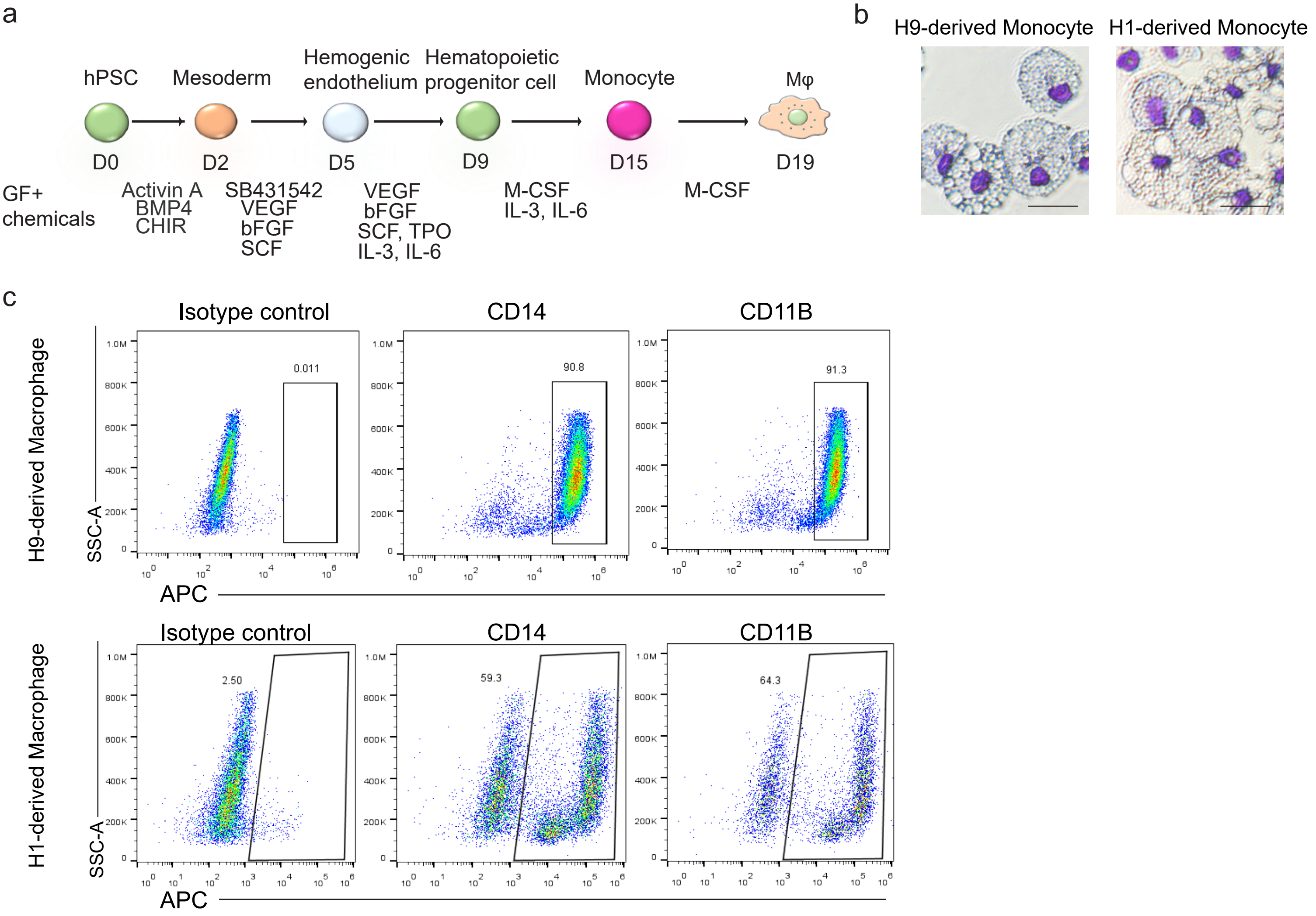

**Extended Data Figure 2. Stepwise differentiation of hPSCs toward macrophages. a,** Scheme of stepwise differentiation of hPSCs toward macrophages. **b,** Swiss-Giemsa staining of hPSC-derived monocytes. Scale bar= 25 μm. **c,** FACS analysis of hPSC-derived macrophages using CD14 and CD11B antibodies.

**Extended Data Figure 3**

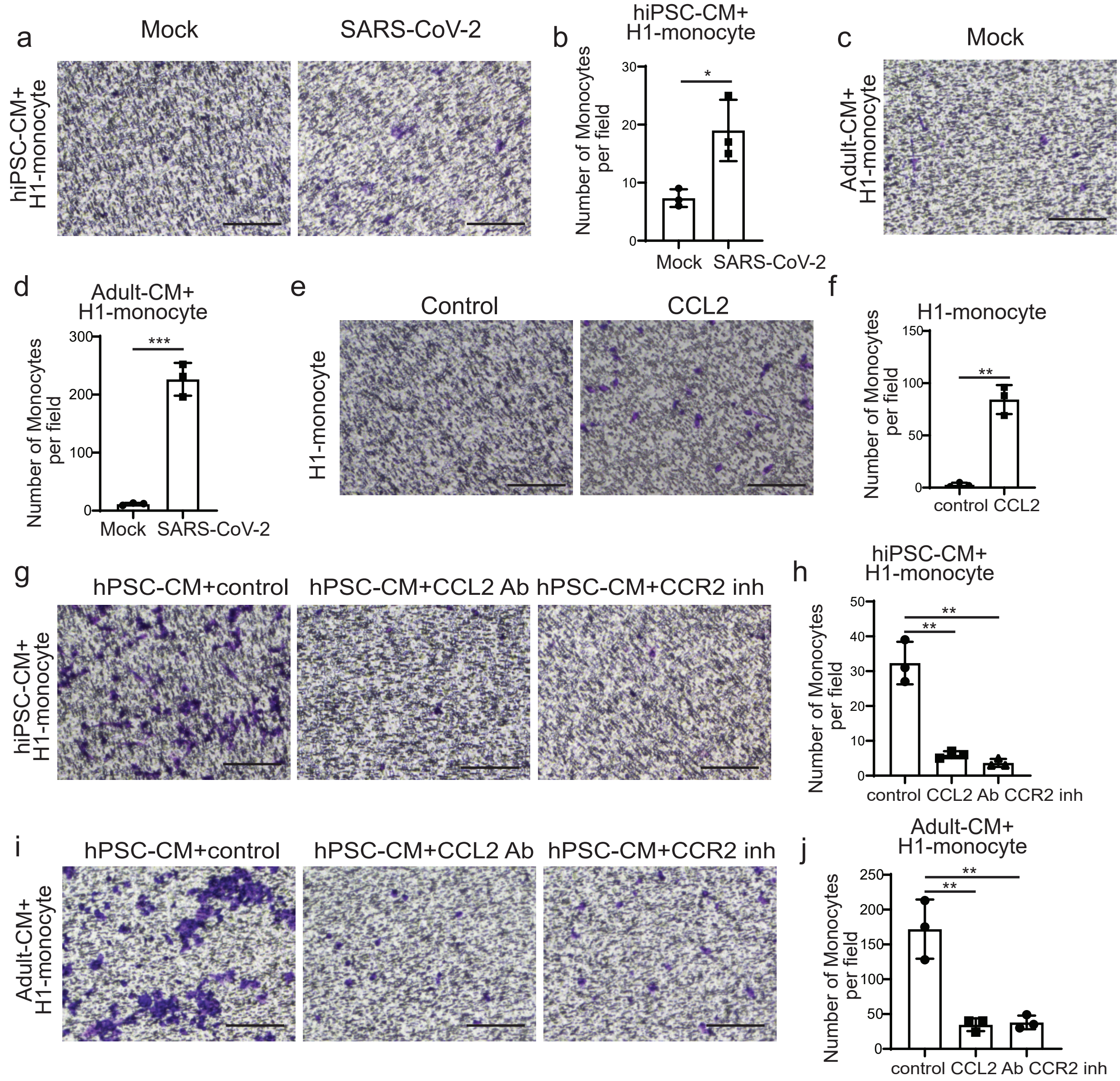

**Extended Data Figure 3. CMs recruit monocytes following SARS-CoV-2 infection through secreting CCL2. a, b,** Phase contract images (**a**) and quantification (**b**) of migrated H1-derived monocytes recruited by hiPSC-derived CMs infected with SARS-CoV-2 virus or mock in the monocyte migration assay. Scale bar= 100 µm. **c, d,** Phase contract images (**c**) and quantification (**d**) of H1-derived monocytes recruited by adult human CMs infected with SARS-CoV-2 virus or mock in the monocyte recruitment assay. Scale bar= 100 µm. **e, f,** Phase contrast images (**e**) and quantification (**f**) of migrated H1-derived monocytes by CCL2 in monocyte recruitment assay. Scale bar= 100 µm. **g, h,** Phase contrast images (**g**) and quantification (**h**) of migrated H1-derived monocytes recruited by hiPSC-derived CMs infected with SARS-CoV-2 virus and treated with CCL2 neutralizing antibody or CCR2 inhibitor: RS504393 in monocyte recruitment assay. Scale bar= 100 µm. **i, j,** Phase contrast images (**i**) and quantification (**j**) of migrated H1-derived monocytes recruited by adult human CMs infected with SARS-CoV-2 virus and treated with CCL2 neutralizing antibody or CCR2 inhibitor: RS504393 in monocyte recruitment assay. Scale bar= 100 µm. N=3 independent biological replicates. Data was presented as mean ± STDEV. *P* values were calculated by unpaired two-tailed Student's t test. *$P < 0.05$, **$P < 0.01$, and ***$P < 0.001$.

## Extended Data Figure 4

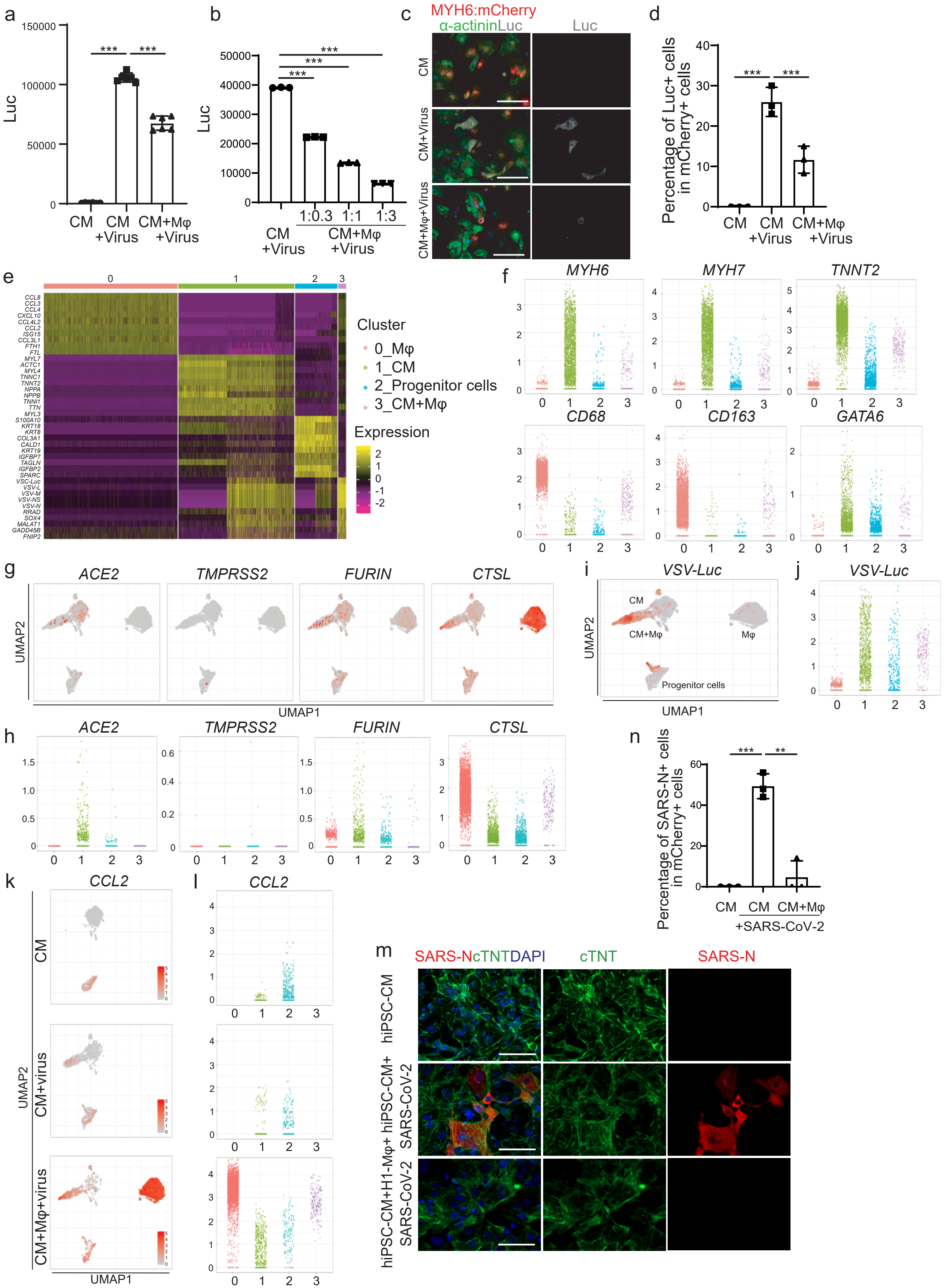

**Extended Data Figure 4. Single cell RNA-seq analysis of immunocardiac co-culture cells upon SARS-CoV-2-entry virus infection. a,** Luciferase activity at 24 hpi of hPSC-derived CMs infected with mock or SARS-CoV-2-entry virus in the presence or absence of macrophages (MOI=0.1). **b,** Luciferase activity at 24 hpi of H9-derived CMs infected with SARS-CoV-2-entry virus and co-cultured with different ratio of macrophages (MOI=0.1). **c, d,** Immunostaining (**c**) and quantification (**d**) of hPSC-derived CMs at 24 hpi with mock or SARS-CoV-2-entry virus in the presence or absence of macrophages (MOI=0.1). **(e)** Heatmap of enriched genes in each cluster of scRNA profiles of the immunocardiac co-culture platform containing hPSC-derived CMs and macrophages upon SARS-CoV-2-entry virus infection. **(f)** Jitter plot of cell type specific markers in the immunocardiac co-culture platform containing hPSC-derived CMs and macrophages upon SARS-CoV-2-entry virus infection. (**g**) UMAP of *ACE2*, *TMPRSS2*, *FURIN*, *CTSL* genes in the immunocardiac co-culture platform containing H9-derived CMs and macrophages upon SARS-CoV-2-entry virus infection. (**h**) Jitter plot of *ACE2*, *TMPRSS2*, *FURIN*, *CTSL* genes in the immune-cardiac co-culture platform containing H9-derived CMs and macrophages upon SARS-CoV-2-entry virus infection. **(i)** UMAP of SARS-CoV-2-entry virus gene in the immunocardiac co-culture platform containing hPSC-derived CMs and macrophages upon SARS-CoV-2-entry virus infection. **(j)** Jitter plot of SARS-CoV-2-entry virus gene in the immunocardiac co-culture platform containing hPSC-derived CMs upon SARS-CoV-2-entry virus infection. (**k**) UMAP analysis of *CCL2* in H9-derived CMs infected with mock (CM) or SARS-CoV-2-entry virus (CM+virus) and the virus-immunocardiac co-culture platform containing H9-derived CMs and H9-derived macrophages infected with SARS-CoV-2-entry virus (CM+macrophage+virus). (**l**) Jitter plot of *CCL2* in H9-derived CMs infected with mock (CM) or SARS-CoV-2-entry virus (CM+virus) and the virus-immunocardiac co-culture platform containing H9-derived CMs and

H9-derived macrophages infected with SARS-CoV-2-entry virus (CM+macrophage+virus). Immunostaining (**m**) and quantification (**n**) of SARS-$N^+$ cells in $cTNT^+$ hiPSC-derived CMs at 24 hpi with mock or SARS-CoV-2 in the presence or absence of H1-derived macrophages (MOI=0.1). N=3 independent biological replicates. Data was presented as mean ± STDEV. *P* values were calculated by unpaired two-tailed Student's t test. $^{**}P < 0.01$, and $^{***}P < 0.001$.

**Extended Data Table 1. Antibodies used for immunocytochemistry and intracellular flow cytometric analysis.**

| Usage | Antibody | Clone # | Host | Catalog # | Vendor | Dilution |
|---|---|---|---|---|---|---|
| FACS | APC anti-human CD11b Antibody | Monoclonal | Rat | #101212 | Biolegend | 1:100 |
| FACS | APC anti-human CD14 Antibody | Monoclonal | Mouse | #301808 | Biolegend | 1:100 |
| Immunocytochemistry | Sarcomeric α-actinin | Polyclonal | Rabbit | #ab137346 | Abcam | 1:500 |
| Immunocytochemistry | Recombinant Anti-Firefly Luciferase antibody | EPR17790 | Rabbit | #ab185924 | Abcam | 1:100 |
| Immunocytochemistry | SARS-CoV/SARS-CoV-2 Nucleocapsid Antibody | #001 | Rabbit | #40143-R001 | SinoBiological | 1:500 |
| Immunocytochemistry | Donkey anti-Mouse IgG (H+L) Highly Cross-Adsorbed Secondary Antibody, Alexa Fluor 488 | Polyclonal | Donkey | #A-21202 | Thermo Fisher Scientific | 1:500 |
| Immunocytochemistry | Donkey anti-Mouse IgG (H+L) Highly Cross-Adsorbed Secondary Antibody, Alexa Fluor 594 | Polyclonal | Donkey | #A-21203 | Thermo Fisher Scientific | 1:500 |
| Immunocytochemistry | Donkey anti-Rabbit IgG (H+L) Secondary Antibody, Alexa Fluor 594 conjugate | Polyclonal | Donkey | #A-21207 | Thermo Fisher Scientific | 1:500 |

| | | | | | | |
|---|---|---|---|---|---|---|
| Immunocytochemistry | Donkey anti-Rabbit IgG (H+L) Secondary Antibody, Alexa Fluor 647 conjugate | Polyclonal | Donkey | #A-31573 | Thermo Fisher Scientific | 1:500 |
| Immunocytochemistry | Donkey anti-Mouse IgG (H+L) Secondary Antibody, Alexa Fluor 647 | Polyclonal | Donkey | #A-31571 | Thermo Fisher Scientific | 1:500 |

**Extended Data Table 2. Primers used for qRT-PCR.**

| Primer name | Sequence |
|---|---|
| *ACTB*-Forward | *CGTCACCAACTGGGACGACA* |
| *ACTB*-Reverse | *CTTCTCGCGGTTGGCCTTGG* |
| *SARS*-*CoV-2-TRS-L* | *CTCTTGTAGATCTGTTCTCTAAACGAAC* |
| *SARS*-*CoV-2-TRS-N* | *GGTCCACCAAACGTAATGCG* |
| *cTNT*-Forward | *TTCACCAAAGATCTGCTCCTCGCT* |
| *cTNT*-Reverse | *TTATTACTGGTGTGGAGTGGGTGTGG* |
